# Investigating the relationship between inbreeding and life expectancy in dogs: mongrels live longer than pure breeds

**DOI:** 10.7717/peerj.15718

**Published:** 2023-07-19

**Authors:** Fernando Mata, Andreia Mata

**Affiliations:** 1 Center for Research and Development in Agrifood Systems and Sustainability, Instituto Politécnico de Viana do Castelo, Viana do Castelo, Portugal; 2 Department of Biosciences, Durham University, Durham, United Kingdom

**Keywords:** Pure breed dog, Cross-bred dog, Mongrel dog, Inbreeding coeficient, Lifespan, Dog, Survivability, Dog welfare, Genetic illness, Dog breeding

## Abstract

This study aimed to investigate the establishment of relationship between inbreeding and life expectancy in dogs. A dataset of *N* = 30,563 dogs sourced from the VetCompass™ Program, UK was made available by the Royal Veterinary College, University of London, containing information about breed and longevity and was subject to survival analysis. A Cox regression proportional hazards model was used to differentiate survivability in three groups of dogs (mongrel, cross-bred and pure breed). The model was found highly significant (*p* < 0.001) and we found that mongrel dog had the highest life expectancy, followed by cross-bred dogs with only one purebred ancestor and purebred dogs had the lowest life expectancy. A second Cox regression was also found highly significant (*p* < 0.001) differentiating the lifespan of different dog breed and correlating positively the hazard ratio and the Genetic Illness Severity Index for Dogs (GISID). The results show that survivability is higher in mongrel dogs followed by cross-bred with one of the ancestor only as a pure breed, and pure breed dog have the highest morbidity level. Higher morbidity is associated with higher GISID scores, and therefore, higher inbreeding coefficients. These findings have important implications for dog breeders, owners, and animal welfare organizations seeking to promote healthier, longer-lived dogs.

## Introduction

Dogs have been our faithful companions for thousands of years, their breeding and genetics have been the subject of growing concern in recent times. Dogs, the human’s best friends, have been domesticated from the wolf during the late Pleistocene more than 15,000 years ago (Horard-Herbin, Tresset & Vigne, 2014). Since the beginning of this process, we have understood that dogs could be useful to human activities in many ways, such as hunting, guarding, herding, waste disposal, warfare, entertainment, pest control, transport, clothing, and even food (Janssens et al., 2018). Having so many potential utilitarian roles led to specialization, and humans started to shape dogs accordingly. More recently, in the past 200 to 300 years shape standards were envisaged, and dog’s morphology was manipulated through closed population breeding (Axelsson et al., 2021). It was however, during the late 19th century, in Victorian Britain, that dog breeding standards started to be implemented, and the pedigree and pure blood concepts reached momentum (Worboys, Strange & Pemberton, 2018) by which modern dog had been ‘invented’.

The modern dog has a multitude of shapes, sizes, colors, and hair types, but also behaviors and personalities, adapted to human needs. The closed population breeding of dogs is a modern practice aiming at the fixation of traits of interest (Axelsson et al., 2021). This bottlenecking of gene flow has also, however, undesirable consequences, as each individual carries deleterious genes with the potential to cause harm and affect fitness and health of dogs (Mabunda et al., 2022). Most of these genes are recessive and can affect the phenotype in homozygosity only. Inbreeding decreases the genetic load and creates in the descendants’ genome long homozygous regions, increasing the potential for deleterious genes to express themselves (Bosse et al., 2019).

The deleterious defects in pure-breed dogs have long ago been identified, and the number of problems identified is growing. Hodgman (1963) identified thirteen conditions, having highlighted the most important being hip dysplasia, patella luxation, entropion, retinal atrophy, and the elongated soft palate. More recently Asher et al. (2009) and Summers et al. (2010), in two companion papers, identified almost 400. At present, the Online Mendelian Inheritance in Animals (OMIA) database, Nicholas & Tammen (2023) identifies 856 trait disorders in dogs.

The lifespan of dogs in relation to breeds and types has been presented and discussed (Patronek, Waters & Glickman, 1997; Salvin et al., 2012) and very recently Teng et al. (2022) produced lifetables based on a large UK population above 30,000 dogs. The enormous diversity of dog breeds, ranging between the 1 kg Chihuahua and the 75 kg Saint Bernard, the 30 cm of the Dachshund and the 85 cm of the Great Dane, determines some variation in the different breeds lifespan (Fleming, Creevy & Promislow, 2011). Several factors of variation have been identified by the different epidemiological studies investigating lifespan in dogs: weight (Adams et al., 2010), neutering status (Moore et al., 2001), breed (Teng et al., 2022), and breed purity (Proschowsky, Rugbjerg & Ersbøll, 2003).

Asher et al. (2009) and Summers et al. (2010) in their two companion papers introduced the Generic Illness Severity Index for Dogs (GISID), where the severity of disorders is sensibly scored. In addition, the authors provide an estimate of these scores for the most common breeds in the UK and also classify the different disorders (inherited defects) as conformation related (C), conformation exacerbated (CD), and not previously linked to conformation (D).

The present study used the lifespan data reported by Teng et al. (2022) to relate with the GISID scores obtained by Asher et al. (2009) and Summers et al. (2010). To investigate the establishment of a relationship between inbreeding and life expectancy in dogs. Understanding of such relationship will be helpful in the scientific management of dog breeding for achieving aparent health and welfare of dogs.

## Materials & Methods

Data are open access (CC BY 4.0) and were retrieved from the Royal Veterinary College, University of London, repository (O’Neill, 2022). The sample includes all dogs under primary veterinary care at clinics participating in the VetCompass™ Program, UK during 2016 (*i.e.,* dogs with at least one clinical record in 2016) (Teng et al., 2022). The dog breeds recognized by any of the Kennel Club (KC), the American Kennel Club, and the Australian National Kennel Council were considered purebred, while all others were considered crosses. Crosses with ancestors not belonging to a recognized breed were considered mongrels. A total of *N* = 30,563 entries were found in the original dataset, which after data cleansing and elimination of outliers, resulted in a sample of *N* = 30,470 dogs (*n* = 2,406 mongrel, *n* = 3,962 crossbred, and *n* = 24,102 pure breed).

Outliers were identified after data transformation for a standard normal distribution (a normal distribution with mean with value zero and standard deviation with value one), and when their z score was above 3 or below -3 standard deviations. Data entered a Cox-regression proportional hazards model for survival analysis with ‘Lifespan’ as the ‘time to event’ variable, and ‘Type of Dog’ (levels: ‘Mongrel’, ‘Cross Bred’, and ‘Pure Breed’) as the factor to analyze. There were no censored entries, and the ‘event’ is ‘Age at Death’ (years). An ANOVA with a least significant difference (LSD) test as *post hoc* was also used to differentiate means between ‘Type of Dog’.

The different pure breed dogs were then compared with the inherited defects in pedigree dogs, namely the disorders related (Asher et al., 2009), and not related (Summers et al., 2010) to breed standards. At this stage, some breeds in the original dataset were eliminated from analysis once to compare with Asher et al. (2009) and Summers et al. (2010) as to consider the breeds reported by these authors. The new dataset comprises *N* = 19,466 dogs distributed within breeds as shown in Table 1.

**Table 1:** Dog breeds with number (*n*) used for Cox-regression proportional hazards model for survival analysis.

Breed	*n*	Breed	*n*
Alaskan Malamute	50	Hungarian Vizsla	44
Basset Hound	76	Irish Setter	39
Beagle	171	Labrador	2,501
Bichon Frise	338	Lhasa Apso	282
Border Collie	1,018	Miniature Schnauzer	211
Border Terrier	294	Poodle Miniature	102
Boxer	831	Poodle Standard	84
Bull Mastif	143	Poodle Toy	56
Bull Terrier	435	Pug	196
Bulldog	501	Rhodesian Ridgeback	68
Cairn Terrier	107	Rottweiler	505
Cavalier King Charles Spaniel	1,004	Scottish Terrier	201
Chihuahua	405	Shar Pei	149
Cocker Spaniel	1,063	Shetland Sheep Dog	61
Collie (Rough)	84	Shih Tzu	635
Dalmatian	149	Springer Spaniel	800
Doberman	148	Staffordshire Bull Terrier	2,344
Dogue de Bordeaux	152	Tibetan Terrier	71
German Shepherd	1,096	Weimaraner	129
German Short-haired Pointer	41	West Highland Terrier	1,103
Golden Retriever	511	Whippet	125
Great Dane	83	Yorkshire Terrier	1,060

The number of identified disorders in the different breeds (inherited defects), were divided by the average scores obtained by the application of the GISID (Asher et al., 2009; Summers et al., 2010) for C, CD, D, and Total, to obtain the variables ‘Rate C’, ‘Rate D’, ‘Rate CD’ and ‘Rate Total’ respectively. These new variables giving an average score per disorder entered a Cox-regression proportional hazards model for survival analysis with ‘Lifespan’ as the ‘time to event’ variable, ‘Breed’ as a factor, and the ‘Rate’ variables as covariates. Again, there were no censored entries, and the event considered is ‘Age at Death’ (years).

The models were tested *via* the −2 Log likelihood test and their parameters *via*, the Wald test. A cumulative survival plot was also produced for the first model.

Data were initially entered in a spreadsheet (Microsoft Excel for Microsoft 365 MSO, version 2204 Build 16.0.15128.20240, 64-bit) for cleansing and outlier detection and elimination. Descriptive statistics were also produced with this software. The Cox regression proportional hazards models were produced with the statistical package IBM Corp. SPSS Statistics, Armonk, NY, USA. Version: 28.0.1.1 (15).

## Results

The descriptive statistics of the variables entered in the first model are shown in Table 2. The ANOVA test was highly significant (*F* = 211.84, 2df, *p* < 0.001) and all the means were significantly different (*p* = 0.032) between ‘Pure Breed’ and ‘Cross Bred’, and *p* < 0.001 for the other comparisons. The ANOVA table is presented as Table 3.

**Table 2:** Descriptive statistics of the lifespan of the three types of dogs (mongrel, cross-bred, and pure breed).

Statistic	Mongrel	Cross bred	Pure breed
Observations	2,406	3,962	24,102
Minimum	0.008	0.003	0.003
1st Quartile	11.006	8.909	9.126
Median	13.569	12.049	11.723
3rd Quartile	15.198	14.350	13.722
Maximum	20.561	21.884	21.539
Mean	12.761	11.225	11.086
Standard deviation	3.762	4.276	3.729
Mean CI (95% upper)	12.838	11.293	11.110
Mean CI (95% lower)	12.684	11.158	11.062

**Table 3:** Analysis of variance (ANOVA) used to test significant differences of lifespan between types of dog (mongrel, cross-bred, cross breed).

Source	Type III sum of squares	df	Mean square	*F*	Sig.
Corrected Model	6,140.12	2	3,070.06	211.84	<0.001
Intercept	1,733,638.69	1	1,733,638.69	119,626.32	<0.001
Dog group	6,140.12	2	3,070.06	211.84	<0.001
Error	441,531.33	30,467	14.49		
Total	4,294,503.46	30,470			
Corrected Total	447,671.45	30,469			

The first model differentiating mongrel, cross bred and pure breed dogs was found to be highly significant (-2 Log Likelihood 567,623, *χ*^2^ = 595, 2 df, *p* < 0.001) and the parameter was also significant (Wald 585, with 2 degrees of freedom, *p* < 0.01). The full model parameterization is shown in Table 4.

**Table 4:** Cox proportional hazards model, modelling the survivability of three types of dogs (mongrel, cross-bred, and pure breed).

	*β*	Wald	df	*p*-value	*e* ^*β*^	*e*^*β*^ 95% CI	
						Lower	Upper
Cross	−0.177	105.474	1	<.001	0.838	0.810	0.867
Mongrel	−0.494	527.621	1	<.001	0.610	0.585	0.637

**Notes.**

The model is highly significant (−2 Log Likelihood 567,623, *χ*^2^ = 595, 2 df, *p* < 0.001). The parameter ‘Type of dog’ is significant (Wald 585, 2 df, *p* < 0.01). ‘Pure breed’ is the baseline type of dog.

**Figure 1:**
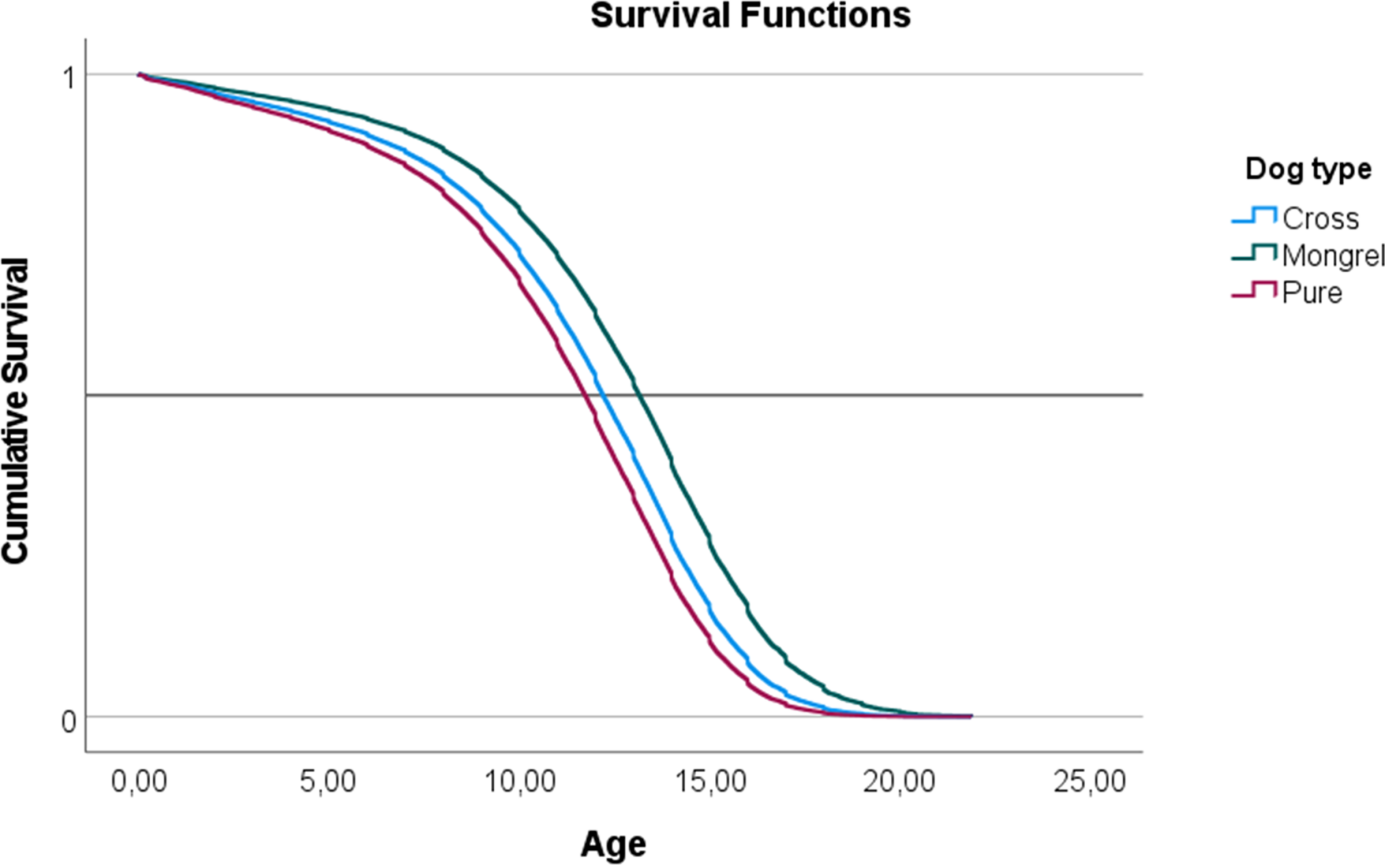
Representation of the survival functions of the Cox-regression proportional hazards model, modeling the survivability of three ‘Types of dog’ (mongrel, cross-bred, and pure breed).

In a Cox proportional hazards model, a negative parameter is indicative of a decrease in the hazard ratio (HR) and an increase in survival in relation to the baseline. The exponential of the parameter (*e*^*β*^) indicates the HR effect size and is interpreted as multiplicative effects on the hazard. Therefore, a cross bred dog has a negative *β*, indicative of a decrease in the HR in relation to a ‘Pure Breed’ of *e*^*β*^ = 0.838 or 17.2%. ‘Mongrel’ dogs also have lower HR in relation to pure breed and the effect size is larger when compared to ‘Cross Bred’. The decrease in the HR in mongrel dogs is 39%. These effects are evident in the survival functions plotted in Fig. 1.

The second model differentiating dog breeds and using the ‘Rates’ calculated from the GISID was also found to be significant (-2 Log Likelihood 335,993, *χ*^2^ = 5,013 with 43 df, *p* < 0.001). The variable ‘Breed’ is significant (Wald 4314, with 42 df, *p* < 0.01), as it is the covariate ‘Total Ratio’ (Wald 64.58, with 1 df, *p* < 0.001). The full model parameterization is shown in Table 5.

**Table 5:** Cox-regression proportional hazards model, modeling the survivability of different breeds of dogs with ‘Rate Total’ as a covariate.

	*β*	Wald	df	*p*-value	Exp(*β*)	Exp (*β*) 95% CI	
						Lower	Upper
Covariate Rate Total	0.162	64.58	1	<0.001	1.175	1.130	1.223
Breed Dogue de Bordeaux	−12.105	44.271	1	<0.001	6^−6^	1.565^−7^	1.960^−4^
Whippet	−2.419	39.009	1	<0.001	0.089	0.042	0.190
Rhodesian Ridgeback	−1.941	24.433	1	<0.001	0.144	0.067	0.310
Staffordshire Bull Terrier	−1.806	40.143	1	<0.001	0.164	0.094	0.287
Tibetan Terrier	0.483	13.633	1	<0.001	1.621	1.255	2.096
Bull Mastiff	0.795	20.258	1	<0.001	2.214	1.566	3.129
Shetland Sheepdog	1.546	61.335	1	<0.001	4.691	3.186	6.907
Pug	1.599	411.244	1	<0.001	4.950	4.241	5.777
Bull Terrier	1.629	102.357	1	<0.001	5.101	3.720	6.995
Poodle Miniature	1.666	38.626	1	<0.001	5.292	3.129	8.950
Poodle Toy	1.798	41.540	1	<0.001	6.037	3.494	10.429
Labrador	1.874	57.486	1	<0.001	6.514	4.013	10.575
West Highland Terrier	1.983	68.114	1	<0.001	7.265	4.536	11.634
Irish Setter	2.103	78.054	1	<0.001	8.187	5.135	13.053
Basset Hound	2.107	127.733	1	<0.001	8.225	5.707	11.853
Cavalier King Chrles Spaniel	2.205	204.593	1	<0.001	9.071	6.705	12.270
German Shorted-Hair Pointer	2.210	65.239	1	<0.001	9.119	5.333	15.592
Chihuahua	2.286	182.833	1	<0.001	9.832	7.059	13.693
Dalmatian	2.329	99.287	1	<0.001	10.270	6.495	16.239
Springer Spaniel	2.423	85.607	1	<0.001	11.278	6.750	18.841
Collie (Rough)	2.431	82.005	1	<0.001	11.376	6.721	19.255
Hungarian Vizsla	2.444	82.177	1	<0.001	11.519	6.791	19.539
Cairn Terrier	2.474	63.367	1	<0.001	11.865	6.453	21.815
Border Collie	2.512	73.124	1	<0.001	12.334	6.935	21.938
Shih Tzu	2.548	83.657	1	<0.001	12.779	7.402	22.059
Poodle Standard	2.642	68.117	1	<0.001	14.036	7.496	26.285
Bichon Frise	2.706	92.274	1	<0.001	14.963	8.615	25.987
German Shepard	2.746	156.512	1	<0.001	15.582	10.134	23.959
Shar Pei	2.813	349.604	1	<0.001	16.662	12.407	22.377
Alaskan Malamute	3.012	132.368	1	<0.001	20.334	12.172	33.969
Cocker Spaniel	3.030	96.623	1	<0.001	20.698	11.312	37.871
Scottish Terrier	3.037	77.295	1	<0.001	20.845	10.591	41.024
Lhasa Apso	3.346	89.761	1	<0.001	28.381	14.205	56.705
Beagle	3.356	112.241	1	<0.001	28.661	15.406	53.320
Golden Retriever	3.488	83.652	1	<0.001	32.730	15.499	69.118
Miniature Schnauzer	3.747	87.736	1	<0.001	42.381	19.351	92.823
Great Dane	3.912	235.326	1	<0.001	50.010	30.337	82.440
Doberman	3.935	162.363	1	<0.001	51.146	27.923	93.682
Boxer	3.983	130.334	1	<0.001	53.691	27.096	106.387
Weimaraner	4.088	99.726	1	<0.001	59.640	26.734	133.048
Rottweiler	4.247	151.288	1	<0.001	69.930	35.540	137.596
Bulldog	4.446	201.831	1	<0.001	85.273	46.178	157.466

**Notes.**

‘Rate Total’ is a calculated rate based on the Genetic Illness Severity Index for Dogs. The model is highly (−2 Log Likelihood 335,993, *χ*^2^ = 5,013 with 43 df, *p* < 0.001). The variable ‘Breed’ is significant (Wald 4,314, with 42 df, *p* < 0.01), as it is the covariate ‘Total Ratio’ (Wald 64.58, with 1 df, *p* < 0.001). Yorkshire Terrier is the baseline breed.

In the second model, the lower the parameter associated with the breed, the higher the HR. Therefore, the breed Dogue de Bordeaux is the breed with the lower HR and higher survivability, while the bulldog has the higher HR and the lower survivability. The breed Yorkshire Terrier is the baseline, therefore negative parameters are associated with lower HR, and positive parameters with higher HR, in relation to this breed. As the model includes forty three breeds any plot of the survival function becomes impossible to read, therefore it is not presented for this model.

The covariate ‘Total Ratio’ indicates that for an additional unit in the ratio, the HR is added by a factor of 17.5% while holding age constant. In other words, the higher the GISID, the higher the death hazard, therefore the lower the survivability. A negative correlation between GISID and survivability becomes, therefore, established within this model.

## Discussion

An increase in dog’s inbreeding coefficients is associated with a lower lifespan. In decreasing order, dogs with longer lifespan are ‘Mongrel’ (mean 12.761 years), followed by ‘Cross Bred’ (11.225 years), and ‘Pure Bred’ (11.086 years). These results were reiterated by the Cox-regression, with a decrease in the HR in relation to pure breed dogs of 17.2% and 39% respectively for cross-bred and mongrel dogs.

Some previous studies have reported this relationship. In a study based on questionnaires filled by members of the Danish Kennel Club and representing a sample of 2,928 dogs, Proschowsky, Rugbjerg & Ersbøll (2003) reported differences between mixed breed (median and interquartile range (IQR) 11 [8, 13]) and several breeds with median varying between 7 and 11. These results were subject to a Kruskal-Wallis test and significant differences were found, however, no *post hoc* tests are reported and, therefore, the statement that differences between pure-breed and cross-bred dogs exist is not robust. Also, a study in Britain (Michell, 1999) using questionnaires, reports that mongrel dogs are between those with larger lifespans (median 14.0). This study, however, does not provide any inferential statistical test and anchors the statement in descriptive statistics only. Patronek, Waters & Glickman (1997) report statistical evidence of significant differences between mixed breeds and pure breeds (medians 8.5 and 6.7 years, respectively), using a large sample (23,535 dogs) of data collected in veterinary hospitals in the USA and Canada. Despite the high credibility of the study, there is no definition of what a mixed breed is; it could eventually be a mongrel, a cross between two pure breeds, or a cross between a mongrel and a pure breed.

Inbreeding is known by agglomerating homozygotic recessive genes in the genome of individuals (Mooney, Yohannes & Lohmueller, 2021), and particularly in pure-breed dogs, it has been shown that higher inbreeding coefficients are associated with higher morbidity (Bannasch et al., 2021; Yordy et al., 2020), due to a larger accumulation of deleterious genes associated with the most common disorders (O’Neill et al., 2014).

The novelty in the present study is the clear differentiation between groups that are not pure breeds. This differentiation allows the definition of three groups with expected different inbreeding coefficients and therefore allows the clear establishment of a relationship between expected inbreeding coefficients and lifespan. Being pure-breed dogs bred in a closed population, they have higher inbreeding coefficients than mongrels; and cross-bred dogs with no more than one pure breed ancestor have, obviously, intermediate inbreeding coefficients. The present study reports results based on a continuous variable allowing the comparison and the report of significantly different means in the three groups of dogs. Previous studies report median values only.

The lifespan differences between breeds have been the objective of study and are well documented, *e.g.*, Bannasch et al. (2021), O’Neill et al. (2013), Teng et al. (2022) and Yordy et al. (2020). The common report in lifespan differences between dog breeds is the size or weight of the breed. Larger dogs are reported to have higher morbidity, *e.g.*, Bannasch et al. (2021), Greer, Canterberry & Murphy (2007), Michell (1999) and O’Neill et al. (2013). This trend is not evident in the present study.

The limitations of this study are associated with the sample. The sample is based on dogs that attended veterinary practices in the UK, and for which both born, and death dates are known. The generalization of the results for other geographies must be done carefully. Also, not all dogs in the UK have records in veterinary practices and many that have records eventually don’t show born and/or death dates. The sample may, therefore, be somehow biased.

Nevertheless, ethical dog breeding must be implemented with respect for dog welfare. Broeckx (2020) revised and elected two points of action: reduce the frequency of disorders and increase genetic diversity. For example in a study (Douglas, Mata & Menem, 2015) of policy comparison between the British Kennel Clube (BKC) and the German Kennel Club, Verband für das Deutsche Hundewesen (VDH) it was demonstrated that it is possible to tackle some of the most common problems faced by pure breeding by reducing the frequency of the disorder. Canine hip scoring can effectively be used in selection programs to reduce the incidence of hip dysplasia. By adopting VDH’s system of mandatory hip scoring of breeding parents and only allowing those with low scores to breed, it was demonstrated that it is possible to reduce faster, hip scores in populations of pedigree dogs when compared with the BKC’s voluntary system.

## Conclusions

It was hypothesized that morbidity in dogs may be associated with inbreeding coefficients. The results of this study confirm the hypothesis, showing that survivability is higher in mongrel dogs followed by cross-bred with one of the ancestor only as a pure breed, and for the last pure breed dogs have the highest morbidity levels. Higher morbidity was associated with higher GISID scores, and therefore, higher levels of homozygotic recessive genes in the genomes of the individuals, and inbreeding coefficients should be reduced for better survivability. Future research may be directed to dog breeding, to decrease inbreeding coefficients and control deleterious genes.

## Additional Information and Declarations

### Data Availability

The following information was supplied regarding data availability: The data is available at the Royal Veterinary College repository: O’Neill, D. 2022. Life tables of annual life expectancy and mortality for companion dogs in the United Kingdom. [Dataset] https://rvc-repository.worktribe.com/output/1558210.
